# Prevalence of treatable and reversible dementias: A study in a dementia outpatient clinic

**DOI:** 10.1590/S1980-57642011DN05010008

**Published:** 2011

**Authors:** Valeska Maria Eboli Bello, Rodrigo Rizek Schultz

**Affiliations:** 1,2 Behavioral Neurology Unit, Department of Internal Medicine, University of Santo Amaro / Unisa, São Paulo SP, Brazil.

**Keywords:** dementia, prevalence, reversible, treatable

## Abstract

Dementia is a syndrome characterized by the development of multiple cognitive deficits and behavioral changes that leads to impairment of functional activities. There are primary degenerative, progressive and irreversible dementias, and other dementias resulting from a progressive but potentially reversible dementia of secondary origin.

**Objective:**

To assess the prevalence of cases with a diagnosis of potentially reversible dementia at a Behavioral Neurology Outpatient Unit.

**Methods:**

A retrospective study based on a review of 340 medical records of patients seen from 1999 to 2009 was conducted. All patients received a thorough diagnostic assessment to verify the etiological hypothesis proposed.

**Results:**

Of the 340 patients seen in the study period, 172 (50.5%) were females and 168 (49.5%) males, 90 patients (26.4%) were under 60 years of age, and 250 (73.6%) were over 60 years of age. Alzheimer’s disease, with 89 cases (26%), followed by vascular dementia with 39 cases (11.47%), were the leading etiological diagnoses. A total of 193 patients had dementia and 37 of these (19.17%) were found to have potentially reversible dementias, distributed as follows: head injury: 15 patients; alcohol-related dementias: 11 patients; meningoencephalitis: 2 patients; hypothyroidism: 2 patients; neurosyphilis: 2 patients; normal pressure hydrocephalus: 2 patients; AIDS: 1 patient; Korsakoff’s syndrome: 1 patient, and Post-anoxic dementia: 1 patient.

**Conclusions:**

A significant number of patients were found to have potentially reversible dementias (19.17%). These data show an urgent need for more extensive diagnostic investigation, and indicate the possibility of reversing some dementias, especially cases detected early.

## Abstract (pt)

Demência é uma síndrome caracterizada pelo desenvolvimento de múltiplos déficits cognitivos e alterações de comportamento, acarretando um prejuízo das atividades funcionais. Há demências degenerativas primárias, progressivas e irreversíveis, e demências que resultam de um progressivo, mas potencialmente reversível processo de origem secundária.

**Objetivo:**

Avaliar a prevalência de pacientes com diagnóstico de demência potencialmente reversível em um ambulatório de neurologia do comportamento.

**Métodos:**

Trata-se de um estudo retrospectivo por meio de revisão de 340 prontuários de pacientes atendidos no período de 1999 a 2009. Todos os pacientes tiveram avaliação diagnóstica criteriosa de acordo com a hipótese etiológica levantada.

**Resultados:**

Dos 340 pacientes atendidos no período, 172 (50,5%) são do sexo feminino e 168 (49,5%) são do sexo masculino, 90 pacientes (26,4%) tinham menos de 60 anos e 250 pacientes (73,6%) tinham mais de 60 anos. Quanto ao diagnóstico etiológico, a maior prevalência foi em relação à doença de Alzheimer com 89 casos (26%), sendo seguida pela demência vascular com 39 casos (11,47%). 193 pacientes tinham demência, sendo que 37 (19,17%) tinham demências potencialmente reversíveis assim distribuídos: traumatismo crânio encefálico: 15 pacientes; demência por álcool: 11 pacientes; hipotiroidismo: 2 pacientes; neurossífilis: 2 pacientes; meningoencefalite: 2 pacientes; hidrocefalia de pressão compensada: 2 pacientes; SIDA: 1 paciente; 1 paciente com síndrome de Korsakoff e 1 paciente com demência pós-anóxia.

**Conclusões:**

Observamos uma parcela significativa de pacientes com demências potencialmente reversíveis (19,17%). Esses dados revelaram a importância da necessidade de uma maior investigação diagnóstica, indicando a possibilidade de prevenção e de reversão de quadros demenciais desde que observados precocemente.

Dementia is a syndrome characterized by the development of multiple cognitive deficits and behavioral changes that leads to impairment of functional activities.^1,2^ The primary degenerative dementias stem from progressive, irreversible neuronal destruction, while reversible dementias are caused by progressive but potentially reversible dementia (PRD) of secondary origin.^3,4^

The following are considered frequent forms of progressive, irreversible dementias (ID): Alzheimer’s disease, vascular dementia, mixed dementia, dementia with Lewy bodies, frontotemporal dementia, Huntington’s disease and Creutzfeldt-Jakob’s disease (CJD).^5^

There are also potentially reversible conditions that may cause or mimic dementia. Among these conditions are brain tumors, head injuries, metabolic changes, thyroid problems and nutritional deficiencies (vitamin B12 being the most common). Toxins, including chronic alcohol abuse, drugs or medication, may cause confusion, cognitive decline and dementia. If detected and treated early, these dementias can be reversed or their progress halted.^6-8^

Malleta proposed the following classification for treatable and potentially reversible dementias:^9^

- Secondary dementias: caused by structural lesions such as normal pressure hydrocephalus, subdural hematoma, brain tumors and post-concussion syndromes; nutritional deficiencies such as vitamin B12 deficiency, folate deficiency, niacin and thiamin deficiency; endocrine disease such as hypothyroidism, hyperthyroidism, adrenal and pituitary gland diseases and insulinoma; vascular diseases and collagenoses such as systemic lupus erythematosus, vasculitis and sarcoidosis; infectious diseases such as meningitis, cerebral abscesses, neurosyphilis, Whipple’s disease, Lyme disease and AIDS; alcoholic dementia and other diseases such as chronic obstructive respiratory disease, sleep apnea syndrome, sleep deprivation, limbic encephalitis, radiation, hypoxia and dialysis.
- Exogenous poisoning and metabolic diseases, such as drug poisoning, hydroelectrolytic changes and chemical poisoning.
- Cognitive disorders due to psychiatric diseases, particularly depression and late-onset schizophrenia.

The aim of the present study was to assess the prevalence of treatable and potentially reversible dementias at a behavioral neurology outpatient unit.

## Methods

All demented and non-demented patients were evaluated at the Behavioral Neurology Unit of the Faculty of Santo Amaro in São Paulo, São Paulo State. The study was approved by the Research Ethics Committee.

A retrospective study was carried out, based on the analysis of records of 340 patients seen at the Behavioral Neurology Outpatients Unit of the Interlagos neighborhood in the city of São Paulo from 1999 to 2009.

Patients treated in this period complaining of cognitive disorders and given the following diagnoses were selected as having potentially reversible dementias and included in the present study:

- Head injury (cranioencephalic trauma - CET).
- Alcohol-related dementia.
- Meningoencephalitis.
- Neurosyphilis.
- Korsakoff’s syndrome.
- Hypothyroidism.
- Acquired immunodeficiency syndrome (AIDS).
- Post-anoxia.
- Normal pressure hydrocephalus.

All patients selected for the study underwent a thorough diagnostic investigation with a detailed history, physical examination, laboratory assessment and imaging studies when necessary.

The diagnosis of dementia was based on the Diagnostic and Statistical Manual of Mental Disorders - Fourth Edition (DSM-lV) criteria for dementia.^2^

All participants were submitted to the Mini-Mental State Examination (MMSE) and to the CERAD (Consortium to Establish a Registry for Alzheimer’s Disease) neuropsychological battery, on which scores were determined for each item and total scale, and performance on the MMSE was adjusted for educational level.^10-12^

The data obtained were analyzed using SPSS for Windows version 13.0.

## Results

Of the 340 patients seen in the Outpatients Unit, 172 (50.5%) were females and 168 (49.5%) were males. A total of 250 (73.6%) of the patients were over 60 years of age, and 90 (26.4%) were under 60 years of age. It should be pointed out that not all patients assessed had a diagnosis of dementia. Some of the patients had normal aging, subjective memory complaints and mild cognitive impairment.

Of all the patients attended in the study period, 193 were found to have dementia and 37 (19.17%) of these had diagnoses of potentially reversible dementia, distributed as per the Table 1.

**Table 1:** Distribution of patients with irreversible dementias (ID), and potentially reversible dementias (PRD), by gender.

	ID (n=156)	PRD (n=37)
Men	48.1%	83.7%
Women	51.9%	16.3%

The two primary diagnoses of dementia found in the study were: Alzheimer’s disease in 89 patients (26%) and vascular dementia in 39 patients (11.4%), with both groups meeting the necessary diagnostic criteria.^13,14^

Of the 156 patients with irreversible dementia, 75 (48.1%) were males with a mean age of 71.6 years (standard deviation of 9.6 years), and 81 (51.9%) were females with a mean age of 75.5 years (standard deviation of 7.8 years). Overall, the mean age of all these patients was 73.6 years (standard deviation of 8.9 years).

Of the 37 patients with potentially reversible dementia, 31 (83.8%) were males with a mean age of 54.5 years, and six (16.2%) were females with a mean age of 58.3 years. A significant difference was found between the two genders (p<0.0001, Chi-Square test). Overall, the mean age of all these patients was 55.2 years (standard deviation of 15.6 years). A total of 23 (62.2%) patients were aged between 21 and 60 years, and the remainder were between 61 and 86 yo. However, no significant difference was found between the two groups (p=0.139, Chi-Square test).

The two most prevalent etiologies were head injury (CET) and alcohol dependence. Among those with head injury (14 males and one female), the average age was 43.7 years whereas for alcohol dependence (9 males and 2 females), the average age was 65.18 years.

## Discussion

Primary degenerative diseases are the main cause of visits to behavioral neurology outpatient units.^15,16^ However, a considerable prevalence (19.17%) of potentially reversible dementia diagnoses was found in the present study. This prevalence is slightly higher than that observed in previous studies.^17,18^

It was found that a great many of these patients were young individuals, evident from the overall average of 55.1 years of age in patients with etiologies of potentially reversible dementias, and from the average of 43.7 years of age for diagnosis of head injury. Most of these patients were therefore still at a productive age when they sought treatment.

Clearly, as meta-analyses have shown, reversibility is more common among younger patients who are less likely to have Alzheimer’s disease or dementia of a vascular cause, and more prone to exotic dementia etiologies, some of which are indeed reversible. Examples include acquired immunodeficiency syndrome dementia complex, hypereosinophilic syndrome, Wilson disease, hypoparathyroidism, adverse effects of valproate therapy in children, the presence of lupus anticoagulant, macroprolactinoma, polycythemia vera, and dural arteriovenous fistula. However, almost all of these conditions tend to occur in patients aged 20 to 60 years and are accompanied by strong indicators in medical history and/or findings on physical examination.^16^ As expected, head injury and alcohol abuse were the diseases commonly observed in younger patients, and potentially can evolve to some kind a deficit such as cognitive impairment. In some communities these conditions are more frequently found with a higher prevalence, as was the case in this study.

There were a wide range of etiologies for these treatable and potentially reversible dementias. However, unlike other studies, infectious etiologies did not predominate.^18^ Neurosyphilis and hydrocephalus occur frequently in some studies, including those conducted in Brazil.^18^

Patients with a diagnosis of vascular dementia were not included in this study, since there is no consensus in the literature concerning the reversibility of this etiology. However, if we were to accept this etiology as being reversible, or rather, if we considered a substantial number of these individuals with a type of vascular dementia that may present a more favorable course and even be partially or totally reversible, the number of patients with possibly reversible dementia could be much higher, since more than 11% of those treated in our Outpatient Unit have vascular dementia.

Notably, the two most prevalent etiologies – head injury and alcohol-related dementia – are related to social issues, and thus the incidence of both could be reduced by social interventions. We also found a greater prevalence of male individuals in relation to these etiologies.

In conclusion, a considerable prevalence of potentially reversible dementias was found in patients seen at the Behavioral Neurology Outpatient Unit. This patient group should be diagnosed early in order to start reversing the clinical picture as early as possible.

In our service, potentially reversible dementias were found to affect predominantly male patients at a productive age. Therefore, greater prevention and more suitable treatment is needed so that, as far as possible, patients’ professional activities are not affected by their cognitive impairments. Diseases, habits or accidents that lead to cognitive disturbances may be avoided by social interventions, and there should be greater concern on the part of society regarding this issue.

## Figures and Tables

**Table 2:** Distribution of patients with potentially reversible dementia by age and gender.

	≤ 60 years	> 60 years	Total
Men	20	11	31
Women	3	3	6
Total	23	14	37

**Table 3:** Etiology and distribution of patients seen with potentially reversible dementia.

Etiology	N	Percentage (%)
Head injury	15	41
Alcohol	11	30
Hypothyroidism	2	5
Normal pressure hydrocephalus	2	5
Neurosyphilis	2	5
Meningoencephalitis	2	5
Acquired immunodeficiency syndrome	1	3
Korsakoff's syndrome	1	3
Post-anoxia	1	3
Total	37	100

## References

[1] Cummings JL (1985). Treatable dementias: differential diagnosis and obstacles to
recognition. Clin Ther.

[2] American Psychiatric Association (1994). Diagnostic and statistical manual of mental disorders - DSM IV.

[3] Kabasakalian A, Finney GR (2009). Reversible dementias. Int Rev Neurobiol.

[4] Gallucci Neto J, Tamelini MG, Forleza OV (2005). Diagnóstico diferencial das
demências. Rev Psiquiatr Clin.

[5] Piccini C, Bracco L, Amaducci L (1998). Treatable and reversible dementias: an update. J Neurol Sci.

[6] Sellal F, Becker H (2007). Démences potentiellement curables. Neurologie.

[7] Sobów T, Wojtera M, Kloszewska I (2007). Potentially reversible dementia in a memory clinic
population. Arch Psychiatr Psychother.

[8] Yousuf RM, Fauzi ARM, Wai KT, Amran M, Akter SFU, Ramli M (2010). Potentially reversible causes of dementia. Int J Collabor Res Int Med Pub Health.

[9] Malleta GJ (1990). The concept of reversible dementia. How nonreliable terminology
may impair effective treatment. J Am Geriatr Soc.

[10] Folstein MF, Folstein SE, McHugh PR (1975). "Mini-mental state". A practical method for grading the cognitive
state of patients for the clinician. J Psychiatr Res.

[11] Bertolucci PHF, Brucki SMD, Campacci SR, Juliano Y (1994). O Mini-Exame do Estado Mental em uma população
geral: impacto da escolaridade. Arq Neuropsiquiatr.

[12] Bertolucci PHF, Okamoto IH, Brucki SMD, Siviero MO, Toniolo Neto J, Ramos LR (2001). Applicability of the CERAD neuropsychological battery to
Brazilian elderly. Arq Neuropsiquiatr.

[13] Mckhann G, Drachman D, Folstein M, Katzman R, Price D, Stadian EM (1984). Clinical diagnosis of Alzheimer's disease: report of the
NINCDS-ADRDA work group under the auspices of the department of health and
human services task force on Alzheimer's disease. Neurology.

[14] Román GC, Tatemichi TK, Erkinjuntti T (1993). Vascular dementia: diagnostic criteria for research studies:
report of NINDS-AIREN International Workshop. Neurology.

[15] Tripathi M, Vibha D (2009). Reversible dementias. Indian J Psychiatry.

[16] Clarfield AM (2003). The decreasing prevalence of reversible dementias: an update
meta-analysis. Arch Intern Med.

[17] Lowenthal DT, Paran E, Burgos L, Williams LS (2007). General characteristics of treatable, reversible, and untreatable
dementias. Am J Geriatr Cardiol.

[18] Takada LT, Caramelli P, Radanovic M (2003). Prevalence of potentially reversible dementias in a dementia
outpatient clinic of a tertiary university-affiliated hospital in
Brasil. Arq Neuropsiquiatr.

